# Putative contribution of CD56 positive cells in cetuximab treatment efficacy in first-line metastatic colorectal cancer patients

**DOI:** 10.1186/1471-2407-10-340

**Published:** 2010-06-30

**Authors:** Raphaël Maréchal, Jef De Schutter, Nathalie Nagy, Pieter Demetter, Arnaud Lemmers, Jacques Devière, Isabelle Salmon, Sabine Tejpar, Jean-Luc Van Laethem

**Affiliations:** 1 Department of Gastroenterology, GI Cancer Unit, Erasme University Hospital, Université Libre de Bruxelles, Brussels, Belgium; 2 Research Fellow of the "Fonds Erasme". Erasme University Hospital, Université Libre de Bruxelles, Brussels, Belgium; 3 Center for Human Genetics, KU Leuven, Leuven, Belgium; 4 Department of Pathology, Erasme University Hospital, Université Libre de Bruxelles, Brussels, Belgium; 5 Laboratory of Experimental Gastroenterology, Department of Gastroenterology and Hepatopancreatology, Erasme University Hospital, Université Libre de Bruxelles, Brussels, Belgium; 6 Department of Gastroenterology and Hepatopancreatology, Erasme University Hospital, Université Libre de Bruxelles, Brussels, Belgium; 7 Digestive Oncology Unit, University Hospital Gathuisberg, Katholieke Universiteit Leuven, Leuven, Belgium

## Abstract

**Background:**

Activity of cetuximab, a chimeric monoclonal antibody targeting the epidermal growth factor receptor, is largely attributed to its direct antiproliferative and proapoptotic effects. Antibody-dependent cell-mediated cytotoxicity (ADCC) could be another possible mechanism of cetuximab antitumor effects and its specific contribution on the clinical activity of cetuximab is unknown.

**Methods:**

We assessed immune cells infiltrate (CD56, CD68, CD3, CD4, CD8, Foxp3) in the primary tumor of metastatic colorectal cancer (mCRC) patients treated with a first-line cetuximab-based chemotherapy in the framework of prospective trials (treatment group) and in a matched group of mCRC patients who received the same chemotherapy regimen without cetuximab (control group). The relationship between intra-tumoral immune effector cells, the K-ras status and the efficacy of the treatment were investigated. We also evaluated *in vitro*, the ADCC activity in healthy donors and chemonaive mCRC patients and the specific contribution of CD56^+ ^cells.

**Results:**

ADCC activity against DLD1 CRC cell line is maintained in cancer patients and significantly declined after CD56^+ ^cells depletion. In multivariate analysis, K-ras wild-type (HR: 4.7 (95% CI 1.8-12.3), p = 0.001) and tumor infiltrating CD56^+ ^cells (HR: 2.6, (95%CI:1.14-6.0), p = 0.019) were independent favourable prognostic factors for PFS and response only in the cetuximab treatment group. By contrast CD56^+ ^cells failed to predict PFS and response in the control group.

**Conclusions:**

CD56^+ ^cells, mainly NK cells, may be the major effector of ADCC related-cetuximab activity. Assessment of CD56^+ ^cells infiltrate in primary colorectal adenocarcinoma may provide additional information to K-ras status in predicting response and PFS in mCRC patients treated with first-line cetuximab-based chemotherapy.

## Background

Cetuximab is a chimeric immunoglobulin G 1 (IgG1) monoclonal antibody (mAb) which binds the epidermal growth factor receptor (EGFR) with high affinity and inhibits ligand binding [1]. Cetuximab is active in chemotherapy resistant metastatic colorectal cancer (mCRC) [2,3] and enhances response rate and progression-free survival (PFS) in first-line therapy in combination with Folfiri and Folfox [4,5]. Clinical studies of cetuximab therapy in mCRC have failed to show a significant correlation between EGFR-staining intensity and patients' response to cetuximab treatment [2,3]. Therefore, identifying molecular markers that can select patients who are likely to benefit from cetuximab is crucial to avoid chemotherapy toxicity and reduce treatment cost. Recently the absence of K-ras mutation appears to be a reliable marker in predicting cetuximab efficacy, both in first-line and in third-line of the anti-EGFR therapies [4-8]. Other factors such as, EGFR amplification [9-11], epiregulin and amphiregulin expression [12], nuclear factor-kB tumor expression [13], PTEN [14], BRAF [15] or PIK3CA [16] were also suggested to predict response to cetuximab but these additional biomarkers require further validation before incorporation into clinical practice.

The activity of cetuximab has largely been attributed to the direct antiproliferative and proapoptotic effects of the antibody. However, another possible mechanism of its antitumor effects is mediated through antibody-dependent cell-mediated cytotoxicity (ADCC). The ADCC mediated through Fc receptors (FcγR) carried by NK cells, macrophages and polymorphonuclear leukocytes, is a well-recognized immune effector mechanism in the antitumor effect of IgG1 [17]. Of these cells, NK cells represent the principal ADCC effector cells [18,19]. Recently, some polymorphisms on genes encoding for activating receptors FcγRIIa and FcγRIIIa were found to affect the clinical efficacy of cetuximab [20,21]. The recruitment of Foxp3-positive regulatory T cells (Treg) into tumor likely represents one of the mechanisms by which malignant cells evade host immune response. The intratumoral density of foxp3 as been reported to be associated with overall survival [22]. Once activated, Tregs can inhibit the function of dendritic cells, NK cells, B cells and other immune cells [23-25] and consequently alter ADCC activity.

Based on the potential value of ADCC in cetuximab activity, we assessed in mCRC patients, the role of peripheral blood mononuclear cells (PBMC) and their CD56+ subpopulation in ADCC activity and we evaluated the relationship between the intratumoral immune cells and the efficacy of first-line cetuximab-based chemotherapy.

## Methods

### Patients and tissue samples

a retrospective study review was conducted from data in our institution's prospectively collected gastrointestinal cancer database. Chemonaive patients with mCRC who underwent surgical resection of their primary tumor and diagnosed with *synchronous *metastases were included in the analysis. We analyzed the primary tumor of 33 chemonaïve mCRC patients treated with first-line cetuximab (Erbitux^®^, Merck, Darmstadt, Germany) containing chemotherapy regimen in the framework of phase II and III studies. Patients received mostly standard FOLFIRI regimen or FOLFOX accordingly to study recommendations [4,26,27]. Thirty-five chemonaïve mCRC patients with *synchronous *metastasis, who underwent resection of the primary tumor before starting a similar chemotherapy regimen that did not contain cetuximab were used as control group. The treatment and the control group were case-matched for the following parameters: sex, age, primary tumor location, tumor stage, performance status, metastatic sites, type of chemotherapy administered and treatment duration.

Formalin-fixed and paraffin-embedded (FFPE) samples of the primary tumor were obtained for immunohistochemical and PCR analysis.

The study was approved by the ethic committee of the Erasme University Hospital and all patients provided written informed consent.

### Clinical evaluation and tumor response criteria

We considered PFS to assess the cetuximab-based chemotherapy efficacy in first line and not OS which is influenced by second and third-lines chemotherapy and liver mets surgery. Tumor response was evaluated by computerized tomodensitometry according to the Response Evaluation Criteria in Solid Tumors (RECIST) [28] and classified in complete response (CR), partial response (PR), stable disease (SD) and progressive disease (PD). The overall best response (OR) was defined as the best response recorded from the start of the treatment until disease progression, recurrence or start of the new therapy. Response was centrally confirmed in the setting of the referenced trials. For the analysis, CR and PR patients were grouped in responders; patients with SD and PD were grouped in non-responder patients.

### Immunohistochemical assessment

Hematoxylin & eosine sections of the tumors were examined by a pathologist for confirmation of the histologic diagnosis and the optimal block donors selected. The FFPE tissues were deparaffinized in xylene, and rehydrated in graded alcohols and water. Colorectal tumor sections were incubated with monoclonal antibodies against CD3, CD4, CD8, CD56, CD68, Foxp3 and EGFR and DAB-chromogen were applied (Dako, Copenhagen, Denmark) (Additional file 1: supplemental Table S1). Appropriate negative and positive controls were used.

### Evaluation of Immunohistochemical variables

The slides were examined independently by two observers (RM, NN) blinded to both clinical and pathologic data. Twenty representative fields of the tumour invasive margin (IM) were chosen from each slide, and the stained cells were counted by means of a 10×10 ocular grid at × 200 magnification (observed area 0.25 mm^2^) using confocal microscopy. For each case, the total number of CD3, CD8, CD4, FOXP3, CD56, CD68 positive cells (representing lymphocytes T (LT), LTCD8, LTCD4, Treg, natural killers (NK) and macrophages, respectively) per square millimeter was calculated. Variations in the enumeration within a range of 5% were re-evaluated and a consensus decision was made.

Expression of EGFR was quantified using a visual grading system based on the extent of staining ( percentage of positive tumor cells graded on scale of 0 to 3: 0, none; 1, 1-30%, 2, 31-60%, 3, > 60% ) and the intensity of staining (graded on a scale of 0 to 3: 0, none; 1, weak staining; 2, moderate staining; 3, strong staining ). Membranous and cytoplasmic staining were evaluated.

### DNA extraction and mutation analysis

Presence of tumor cells (> 75%) in each tumor block was firstly histologically controlled by H & E coloration. Thereafter, DNA was extracted from FFPE samples after macrodissection. The presence of K-ras was determined by allelic discrimination assay on a 7500HT Real Time PCR System. K-ras mutations located within the codon 12 (n = 6) and 13 (n = 1) were screened for. All mutations were confirmed by direct sequencing (8).

### Cell Lines and cell culture

The EGFR overexpressing DLD1 colorectal glandular carcinoma cell line was obtained from the American Type Culture Collection (Manassas, VA, USA). DLD1 has been previously found to carry mutated K-ras. To confirm that DLD1 indeed harbour mutated K-ras alleles, we extracted the corresponding genomic DNA and sequence the K-ras locus. We confirmed that DLD1 display Gly13Asp K-ras mutation (data not shown).The DLD1 cells were maintained in RPMI 1640 (Sigma, St Louis, MO, USA) supplemented with 10% heat-inactivated foetal bovine serum (FBS, Gibico BRL, Grant Island, NY, USA), sodium pyruvate 1%, 100 units/ml penicillin and 100 μ g/ml streptomycin. The expression of EGFR by the DLD1 cell line was confirmed by immunocytochemistry (data not shown)).

### Preparation of human peripheral blood mononuclear cells (PBMC)

PBMC from 5 healthy medication-free donors (3 male, 2 female; mean age: 33.8 years, range:27-48 years) and 5 chemonaïve mCRC patients (2 female, 3 male, mean age 55.4 years, range 49-61 years) were isolated from heparinised peripheral blood on a Ficoll gradient.

### Flow cytometry analysis

Immunophenotyping of PBMCs before and after depletion of CD56+ cells was performed by incubation with appropriate combination of fluorochrome-labeled monoclonal antobodies. Major lymphocyte populations CD3+ , CD3+ CD4+ , CD3+ CD8+ , CD3 - CD16+ 56+ (NK), CD3+ CD16+ 56+ (NKT), CD19+ (B) were determined by two cocktails MoAb: anti-CD45 PerCP, anti-CD3 FITC, anti-CD8 PE, anti-CD4 APC and anti-CD45 PerCP, anti-CD3 FITC, anti-CD16+ 56 PE and CD19 APC. Data acquisition was performed with a FacsCanto flow cytometer and data analysed using BD FacsDiva software (BD Biosciences, Mountain View, CA).

### Purification of effector cells and interleukine-2(IL-2) treatment

Highly depleted PBMC from CD56^+ ^cells (CD56 depleted PBMC), were obtained by magnetic activated cells sorting (MACS) using the system from Miltenyi Biotec according to the manufacturer's instructions. The CD56 depleted PBMC and non depleted PBMC were used separately in ADCC assays. Between 92% to 94% of the CD56^+ ^cells were sorted after MACS depletion. As IL-2 is known to activate PBMC, we tested the effect of human recombinant IL-2 (R & D Systems) on cetuximab-mediated ADCC in CD56 depleted and non depleted PBMC. The two populations were cultured in medium alone or enriched with rhuIL-2 (10 ng/ml) for 18 h prior to use in ADCC assays.

### ADCC assay

5 × 10^4^target cells/well were plated in a 96 flat bottom wells plate in 200 μ l of medium, 24 hours before adding effectors cells. Human PBMC (effector) or IL-2 activated PBMC, were added at different E:T ratios ranging from 20:1 to 2:1 and incubated for 24 h. Cetuximab or polyclonal human IgG (Sigma, St Louis, MO, USA) were added to the individual wells at different concentrations ranging from 0 to100 μ g/ml.

Accordingly to *Heo et al*., cytotoxicity was evaluated using a 3-(4,5 dimethyltiazol-2-yl)-2,5-diphenyltetrazolium bromide (MTT) colorimetric assay [29,30] (additional file 2). Experiments conducted in a preliminary phase to select optimal conditions for the ADCC effect showed that *(a) *target cells were not killed after exposure to cetuximab in the absence of PBMC *(b) *10 μ g/ml was the optimal cetuximab concentration for saturating ADCC assay by PBMC.

### Statistical Analysis

The PFS was estimated by the Kaplan-Meier method and the two groups were compared with by the log-rank test. For all immunohistochemical markers, the cut off for definition of subgroups was the median value. The non- parametric χ^2 ^test and the Fisher's exact tests were carried out as appropriate to compare categorical variables. The Mann-Witney U test was used for the comparison of the treatment and the control groups regarding the tumor infiltrating immune cells and ADCC activity. Correlations were analyzed by Spearman's correlation coefficient test. Multivariate analyses used a step-down procedure based on the likelihood ratio test. A p-value ≤ 0.1 in univariate analysis was required to consider the variable for multivariate analysis. The Two-tailed p < 0.05 was judged to be significant. All analysis were performed with SPSS 10.0 software (SPSS, Chicago, Il).

## Results

### Patients characteristics

The baseline and treatment characteristics of patients from treatment and control group are summarized in table 1. The two groups were similar regarding all variables matched including the proportion of patients treated with the FOLFIRI

**Table 1:** Patients characteristics

Variables	Treatment group (n = 33)	Control group (n = 35)	p-value
Age, median (range)	59 (43-75)	62 (41-78)	0.74

Sex, n			
Male	19	20	
Female	14	15	0.97

ECOG PS, median (range)	0 (0-1)	0 (0-2)	0.37

Primary tumour location			
Right	11	11	
Transverse	1	2	
Left	18	19	
rectum	3	3	0.91

Metastatic sites			
Liver	24	26	
Lymph nodes	7	8	
Lung	2	1	0.98

Chemotherapy regimen			
Folfiri + cetuximab	32	0	
Folfiri	0	33	
Folfox + cetuximab	1	0	0.94
Folfox	0	2	

Overall Best Response			
CR	2	1	
PR	14	15	
SD	6	9	
PD	11	10	0.36

Treatment duration			
months, median	4.9	5.3	0.51

PFS			
Months, median	5.7	5.4	0.52

Abbreviations: NS: non significant, ECOG PS: Eastern Cooperative Oncology Group Performance Status.

### Immune cells infiltrate in primary CRC and treatment efficacy

**Figure 1:**
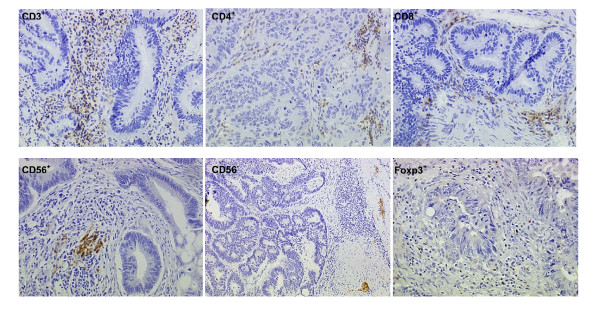
**Sections of colorectal adenocarcinoma with results of the immunostaining: CD3, CD4, CD8, CD56, Foxp3 (magnification × 100)**. The CD56- section (magnification X40) reveals no CD56 positive immune cells (considered as NK negative tumor) but staining of Meisner plexus used as interne positive control.

Positive CD4, CD3, CD8, CD68 and Foxp3 cells were detected in 33/33 (100%) tumor samples (figure 1) and their mean number were similar between the two patient groups (table 2). The number of intratumoral macrophages (CD68^+ ^cells) and Lymphocytes (CD3^+ ^,CD4^+ ^,CD8^+ ^, Foxp3^+ ^cells) were similar between responders and non responders and we found no association with the PFS both in the treatment and the control group (table 3).

**Table 2:** Mean numbers of tumor-infiltrating immune cells in treatment and control groups

Variables	Treatment Group		Control Group		Treatment *vs *Control group
	
	Mean	Median	Mean	Median	p-value
	**cells/mm**^**2**^	**cells/mm**^**2**^	**cells/mm**^**2**^	**cells/mm**^**2**^	
	(SD)	(range)	(SD)	(range)	
CD3+	139 (108)	99 (0-340)	106 (89)	144 (0-410)	0.82
CD4+	33 (27)	42 (0-114)	24 (19)	28 (0-82)	0.21
CD8+	111 (106)	65 (29-331)	126 (101)	68 (28-361)	0.71
CD68+	171 (80)	88 (31-465)	139 (127)	151 (22-385)	0.27
Foxp3+	43 (36)	40 (14-150)	114 (101)	76 (12-228)	0.54
CD56+	6.4	0 (0-18)	5.6	0 (0-15)	0.32

Abbreviations: SD: Standard Deviation

**Table 3:** Univariate and multivariate analysis of factors associated with OR and PFS

UNIVARIATE ANALYSIS				
**Variables**	**OR**		**PFS**	
	
	**HR (95% CI)**	**p-value**	**HR (95%CI)**	**p-value**

**Treatment Group**				
CD3+	0.80 (0.20-3.25)	0.70	0.94 (0.45-1.94)	0.91
CD4+	1.31 (0.32-1.49)	0.36	0.74 (0.35-1.58)	0.41
CD8+	0.86 (0.22-3.58)	0.74	0.84 (0.45-1.72)	0.62
CD56+	7.75 (1.13-42.6)	0.012	2.70 (1.25-5.88)	0.005
CD68+	1.25 (0.79-7.72)	0.55	1.16 (0.55-2.46)	0.69
Foxp3+	0.54 (0.20-3.50)	0.98	0.89 (0.43-1.83)	0.81
K-ras status				
WT vs MT	12.81 (2.15-7.7)	0.008	4.36 (1.77-10.7)	0.0007

**Control Group**				
CD3+	0.91 (0.41-4.11)	0.63	0.84 (0.38-3.98)	0.74
CD4+	1.51 (0.61-2.74)	0.54	0.94 (0.52-2.98)	0.52
CD8+	1.32 (0.59-2.42)	0.31	1.20 (0.59-2.31)	0.76
CD56+	1.21 (0.74-2.07)	0.29	1.11 (0.81-1.56)	0.14
CD68+	0.84 (0.58-2.11)	0.36	0.87 (0.44-3.52)	0.27
Foxp3+	0.66 (0.34-1.92)	0.68	0.71 (0.88-1.56)	0.13

**MULTIVARIATE ANALYSIS**				

Variables	OR		PFS	
	
	HR (95% CI)	p-value	HR (95%CI)	p-value

CD56				
Negative	1		1	
Positive	7.05	0.04	2.62	0.0019
	(1.49-45.76)		(1.14-6.00)	
K-ras				
Mutated	1		1	
Wild Type	11.7	0.013	4.74	0.001
	(1.77-8.33)		(1.8-12.3)	

Abbreviations: CI: Confident interval, MT: mutated, WT: wild type, OR: overall response rate, PFS: Progression-free survival

For CD56+ cells, two distinct immunologic pattern were clearly observed consisting in, either tumors with strongly positive CD56 staining (CD56 positive tumors) or tumors with undetectable CD56 staining (CD56 negative tumors) (figure 1).These two pattern were found both in the treatment and the control groups (table 2) and were compared for OR and PFS and correlated with the K-ras status. In the control group, no difference has been detected in OR and PFS between patients with CD56 negative tumor cells and those with CD56 positive tumor (table 3, figure 1). By contrast, in the treatment group, CD56 positive tumors were more frequent in responder than in non-responder patients (10/16 (63%) *versus *3/19 (18%), p = 0.011) and patients with CD56 positive tumor had a longer PFS than those with CD56 negative one (8.8 months (95% CI: 3.3-13.5) versus 3.9 months (95% CI: 3.1-4.7)) (p = 0.005) (table 3). Neither the CD56 tumor status (r = -0.267, p = 0.134) and the number of CD56^+ ^cells (r = -0.295, p= 0.101) were correlated with the K-ras status of the tumor.

### EGFR expression

as previously observed, no significant correlation was found between EGFR expression, OR rate and PFS.

### K-ras mutation Status and treatment efficacy

K-ras mutations were detected in the tumor of 13/33 (39%) patients from the cetuximab treatment group. Wild type (WT) status was found in 13/16 (81%) responders and in 7/17 (41%) non responders (p = 0.008). WT group had a prolonged PFS (9.2 months (95% CI: 4.9 to 16.4)) as compared to the mutated group (4.5 months (95% CI: 2.6-6.2; p = 0.0007) (table 3).

### Multivariate analysis

CD56+ cells infiltrate and WT K-ras status were independent predictors of OR. Both CD56 negative tumors (Hazard ratio: 2.6 (95% CI: 1.14-6.00); p = 0.019) and K-ras mutations (Hazard ratio: 4.74 (95% CI: 1.8-12.3); p = 0.001) contribute as significant independent negative prognostic factors for the PFS (table 3). Interestingly, in the group of WT K-ras tumors (n = 20), there is a trend for a higher OR and a prolonged PFS in patients with CD56 positive tumor as compared with CD56 negative tumor patients and the whole group (CD56^+ ^and CD56^- ^) (additional file 3, Supplemental Table S2).

### Cetuximab-mediated ADCC in vitro activity is maintained in mCRC patients

CD56^+ ^cells represented a minor fraction (range between 5 and 14%) of the whole PBMC population.

**Figure 2:**
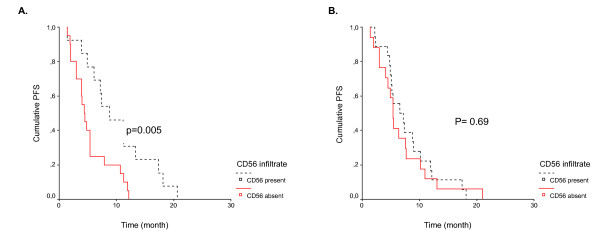
**PFS according to tumor CD56 status in the treatment group (A) and the control group (B)**.

**Figure 3:**
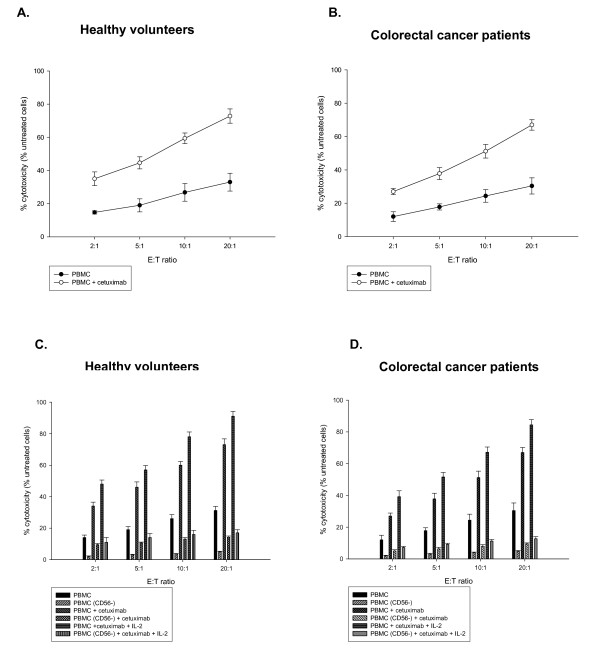
**Cytotoxicity against colorectal cancer cell line mediated by cetuximab**. **A-B: **PBMCs from healthy volunteers (n = 5, mean age 31 years) and from 5 mCRC patients (n = 5) mean age 52 years) were tested for cytotoxicity against DLD1, using four E:T ratio in the absence (PBMCs activity) or presence of cetuximab (10 μ g/ml). Points mean of a triplicate experiment; bars: SD. **C-D: **PBMCs from 5 healthy donors and 5 mCRC were tested for the cytotoxicity in the presence or the absence of cetuximab, for the effect of IL-2 stimulation and CD56^+ ^cells depletion. Columns, mean; bars, SD

Adjunction of cetuximab enhanced the cytotoxicity of PBMC (ADCC) as compared to the PBMC activity (PBMC alone). At the higher E:T ratios of 10:1 and 20:1, cetuximab enhanced tumoral cell lysis, as compared to the baseline activity of PBMC; both in healthy volunteers (p = 0.008 at an E:T ratio of 10:1,from 26% to 60%; and p = 0.009 at an E:T ratio of 20:1, from 30% to 74%) and in mCRC patients (p = 0.001 at an E:T ratio of 10:1,from 19% to 56%; and p = 0.002 at an E:T ratio of 20:1 from 25% to 71%) (figure 2 and 3B). PBMC activity was not increased by the control antibody. Interestingly, PBMC and ADCC activity was similar between mCRC patients and healthy volunteers.

### CD56^+ ^cells are responsible for cetuximab-mediated ADCC in vitro activity and its majoration by IL-2

While CD56^+ ^cells represent a minor fraction of the PBMC, depletion of CD56^+ ^cells significantly reduced the ADCC activity at the different E:T ratio (p < 0.05 at all E:T ratios) even after IL-2 stimulation (figure 3C, 3D) underlying the major role of this immune cells in ADCC activity.

## Discussion

One of the most important mechanisms of cetuximab and panitumumab, the monoclonal antibodies that target the EGFR, is through the inhibition of the EGF receptor/ligand interaction. The other pathway through which cetuximab only, as an IgG1, may exert its antitumor effect is ADCC [12,14]. Several *in vitro *and *in vivo *studies have shown that this chimeric IgG1 mAb binds to the antigen, the EGFR, on the surface of tumor cells while its Fc portion binds to the immune effector cells through FCγ R. Consequently, this binding activates the immune cells leading to tumor cells killing. ADCC activity of mAbs has been well described for trastuzumab, a human IgG1 anti human EGFR 2 (Her-2) antibody and for rituximab, a chimeric IgG1 mAb for B-cell differentiation antigen CD20 [31-33].

The array of cellular effectors potentially exerting ADCC includes the CD3^- ^CD56^+ ^NK lymphocytes [34] but also CD3^+ ^CD16^+ ^T-cell subset [35], CD16^+ ^CD33^+ ^macrophages [36] and CD16^+ ^granulocytes [37]. Among the mononuclear cells (MNC), the NK cells (CD3^- ^CD56 ^+ ^) play a predominant role in the ADCC through low affinity type II (Fc*γ *RIIc) and type IIIA (Fc*γ *RIIIa) Fc receptors present on their surface [38,39]. The polymorphonuclear cells are also able to induce ADCC by the engagement of their CD32 receptor but needs higher concentration levels of antibody compare with the MNC.

Since only less than 50% of WT-Kras mCRC patients seem to benefit from cetuximab-based treatment, it may be highly relevant to identify other biomarkers than K-ras status to predict cetuximab efficacy. To this end, the evaluation of the ADCC actors as putative surrogate markers of cetuximab-based therapy seems to be attractive. As there is still debate whether ADCC plays a role in metastatic cancer patients who mostly have suppressed immune function, we choose to evaluate cetuximab-mediated ADCC activity in front-line chemonaïve mCRC patients. We only used an EGFR^+ ^cell line and no control EGFR^- ^cell since the activation of ADCC activity by cetuximab requires the expression of its target, the EGFR [40]. Our functional assay provided evidences that CD56^+ ^cells are the main effectors of cetuximab-mediated ADCC in mCRC patients. Furthermore, the PBMC of mCRC were capable to initiate ADCC comparable to healthy donors suggesting that their function is maintained. Furthermore, we observed a significant *in vitro *ADCC activity despite the mutated K-ras status of the DLD1 cell line. Our results are thus in accordance with a recent report showing that cetuximab-related ADCC is irrespective of the K-ras status [40]. This property could be exploited particularly in patients with K-ras*-*mutated tumors, who otherwise have a low probability of responding to cetuximab [4,6,7]. In this way, approaches to enhance ADCC activity, such as immunostimulation and new generation of EGFR-directed mAbs, may be promising in the management of this subset of patients.

Whether ADCC contributes to the clinical efficacy of cetuximab, remains to be determined. Indeed, cetuximab and panitumumab displayed similar clinical activity despite the fact that panitumumab is a fully human IgG2 monoclonal antibody with low ability to recruit immune cells compared to IgG1. The adding value of cetuximab-mediated ADCC might deserve direct comparison between cetuximab and panitumumab. On the other hand, two studies have demonstrated that FCγ R gene polymorphisms (Fc R3A-V158F and Fc R2a-H131R) are associated with clinical outcome (response rate and PFS) in mCRC patients treated with cetuximab administered in combination with irinotecan or as a single-agent [20,21]. These findings may support the contribution of ADCC in cetuximab efficacy. In this line, it would be helpful to identify which surrogate markers may help us to predict this contribution to efficacy.

Complementary to our in vitro assay, we observed that the OR and PFS were significantly higher in patients with CD56 positive tumors as compared with CD56 negative tumors. This was not observed in a matched control group. Nevertheless, only evaluation in prospective randomized trials may confirm that CD56 tumor status is specifically predictive for ADCC contribution and not only prognostic.

When adjusted for K-ras status in multivariate model, the CD56 tumor status remains an independent marker of response to treatment (OR) and of better PFS and could be therefore of additional interest in predicting enhanced cetuximab activity in the WT population.

Our data raise additional questions regarding the role of NK cells. The phenotype and properties of tumor-infiltrating NK cells seems to be different from that of peripheral NK cells. Peripheral NK cells are CD3^- ^CD56^low ^CD16^+ ^while intratumoral NK cells are usually CD3^- ^CD56^bright ^CD16 ^- ^and unable to do ADCC [41,42]. The number of intratumoral CD56^+ ^cells was found to be low (mean number: 6.4/mm^2 ^and 5.6/mm^2 ^in the treatment and the control group respectively) and it may be thus questionable how this small number can truly impact on CTX efficacy. However, while the circulating CD56^+ ^cells represent a minor fraction of PBMC, we observed *in vitro *that their low number does not preclude for ADCC intensity. Additionally, some studies have evidenced significant immune cells recruitment into the tumors after trastuzumab or rituximab based therapy [38,43,44] even when trastuzumab was associated with immunosuppressive chemotherapy [38]. This recruitment was more significant in tumors exhibiting higher in situ infiltration lymphocytes infiltrate at the baseline [44]. All together, these data suggest that intratumoral CD56^+ ^cells might be a surrogate marker of subsequent recruitment.

## Conclusions

In summary, the presence of tumor-infiltrating CD56^+ ^cells is an independent predictor for PFS and OR in mCRC patients treated with first line cetuximab based-chemotherapy. This suggests that ADCC, mainly through its central effector, the NK cells, could influence response to cetuximab-based chemotherapy. The exact relationship between TIL of the primary cancer and peripheral NK cells activity should be further explored, notably in assessing the role of ADCC in the adjuvant setting in patients treated with cetuximab.

## Competing interests

The authors declare that they have no competing interests.

## Authors' contributions

RM designed the study, performed IHC and manual analysis, *in vitro *experiments, statistical analysis and drafted the manuscript, JDS performed PCR analysis, NN performed IHC and manual evaluation, PD performed IHC and manual evaluation, AL designed the study and performed *in vitro *experiments, JD, designed the study and drafted the manuscript, IS designed the study and drafted the manuscript, ST designed the study and drafted the manuscript, JLVL designed the study and drafted the manuscript. All authors read and approved the final manuscript.

## Pre-publication history

The pre-publication history for this paper can be accessed here:

http://www.biomedcentral.com/1471-2407/10/340/prepub

## Supplementary Material

Additional file 1 **Supplemental Table S1**. Summary of the immunohistochemistry. Click here for file

Additional file 2 **Description of the MTT assay**. Click here for file

Additional file 3 **Supplemental Table S2**. Cetuximab-based chemotherapy efficacy in WT tumors based on the presence or absence of tumor infiltrating CD56+ cells. Click here for file

## References

[B1] Harding J Burtness B Cetuximab: an epidermal growth factor receptor chemeric human-murine monoclonal antibody Drugs Today 2005 41 107 27 10.1358/dot.2005.41.2.882662 15821783

[B2] Cunningham D Humblet Y Siena S Cetuximab monotherapy and cetuximab plus irinotecan in irinotecan-refractory metastatic colorectal cancer N Engl J Med 2004 351 337 345 10.1056/NEJMoa033025 15269313

[B3] Saltz LB Meropol NJ Loehrer PJ Sr Phase II trial of cetuximab in patients with refractory colorectal cancer that expresses the epidermal growth factor receptor J Clin Oncol 2004 22 1201 1208 10.1200/JCO.2004.10.182 14993230

[B4] Van Cutsem E Lang I D'haens G KRAS status and efficacy in the first-line treatment of patients with metastatic colorectal cancer (mCRC) treated with FOLFIRI with or without cetuximab: The CRYSTAL experience N Eng J Med 2009 2 1408 17 10.1056/NEJMoa0805019

[B5] Bokemeyer C Bondarenko I Makhson Fluorouracil,leucovorin, and oxaliplatin with and without cetuximab in the first-line treatment of metastatic colorectal cancer J Clin Oncol 2009 27 663 71 10.1200/JCO.2008.20.8397 19114683

[B6] Lièvre A Bachet JB Boige V KRAS mutations as an independent prognostic factor in patients with advanced colorectal cancer treated with cetuximab J Clin Oncol 2008 26 374 9 10.1200/JCO.2007.12.5906 18202412

[B7] Lièvre A Bachet JB Le Corre D KRAS mutation status is predictive of response to cetuximab therapy in colorectal cancer Cancer Res 2006 66 3992 5 10.1158/0008-5472.CAN-06-0191 16618717

[B8] De Roock W Piessevaux H De Schutter J KRAS wild-type state predicts survival and is associated to early radiological response in metastatic colorectal cancer treated with cetuximab Ann Oncol 2008 19 508 15 10.1093/annonc/mdm496 17998284

[B9] Moroni M Veronese S Benvenuti S Gene copy number for epidermal growth factor receptor (EGFR) and clinical response to anti-EGFR treatment in colorectal cancer: A cohort study Lancet Oncol 2005 6 279 286 10.1016/S1470-2045(05)70102-9 15863375

[B10] Personeni N Fieuws S Piessevaux H Clinical usefulness of EGFR gene copy number as a predictive marker in colorectal cancer patients treated with cetuximab: A fluorescent in situ hybridization study Clin Cancer Res 2008 14 5869 5876 10.1158/1078-0432.CCR-08-0449 18794099

[B11] Sartore-Bianchi A Moroni M Veronese S Epidermal growth factor receptor gene copy number and clinical outcome of metastatic colorectal cancer treated with panitumumab J Clin Oncol 2007 25 3238 45 10.1200/JCO.2007.11.5956 17664472

[B12] Khambata-Ford S Garrett CR Meropol NJ Expression of epiregulin and amphiregulin and K-ras mutation status predict disease control in metastatic colorectal cancer patients treated with cetuximab J Clin Oncol 2007 25 3230 7 10.1200/JCO.2006.10.5437 17664471

[B13] Scartozzi M Bearzi I Pierantoni C Nuclear factor-kB tumor expression predicts response and survival in irinotecan-refractory metastatic colorectal cancer treated with cetuximab-irinotecan therapy J Clin Oncol 2007 25 3930 3935 10.1200/JCO.2007.11.5022 17761976

[B14] Frattini M Saletti P Romagnani E PTEN loss of expression predicts cetuximab efficacy in metastatic colorectal cancer patients Br J Cancer 2007 97 1139 45 10.1038/sj.bjc.6604009 17940504 PMC2360431

[B15] Di Nicolantonio F Martini M Molinari F Wild-type BRAF is required for response to panitumumab or cetuximab in metastatic colorectal cancer J Clin Oncol 2008 26 5668 70 10.1200/JCO.2008.18.0786 19001320

[B16] Loupakis F Pollina L Stasi I PTEN expression and KRAS mutations on primary tumors and metastases in the prediction of benefit from cetuximab plus irinotecan for patients with metastatic colorectal cancer J Clin Oncol 2009 27 2622 9 10.1200/JCO.2008.20.2796 19398573

[B17] Weiner GJ Monoclonal antibody mechanisms of action in cancer Immunol Res 2007 39 271 278 10.1007/s12026-007-0073-4 17917071

[B18] Kurai J Chikumi H Hashimoto K Antibody-dependent cellular cytotoxicity mediated by cetuximab against lung cancer cell lines Clin Cancer Res 2007 13 1552 61 10.1158/1078-0432.CCR-06-1726 17332301

[B19] Hara M Nakanishi H Tsujimura K Interleukin-2 potentiation of cetuximab antitumor activity for epidermal growth factor receptor-overexpressing gastric cancer xenografts through antibody-dependent cellular cytotoxicity Cancer Sci 2008 99 1471 8 10.1111/j.1349-7006.2008.00821.x 18422755 PMC11159884

[B20] Zhang W Gordon M Schultheis AM FCGR2A and FCGR3A polymorphisms associated with clinical outcome of epidermal growth factor receptor expressing metastatic colorectal cancer patients treated with single-agent cetuximab J Clin Oncol 2007 25 3712 8 10.1200/JCO.2006.08.8021 17704420

[B21] Bibeau F Lopez-Crapez E Di Fiore F Impact of Fc{gamma}RIIa-Fc{gamma}RIIIa polymorphisms and KRAS mutations on the clinical outcome of patients with metastatic colorectal cancer treated with cetuximab plus irinotecan J Clin Oncol 2009 27 1122 1129 10.1200/JCO.2008.18.0463 19164213

[B22] Salama P Phillips M Grieu F Tumor-infiltrating FOXP3+ T regulatory cells show strong prognostic significance in colorectal cancer J Clin Oncol 2009 27 186 92 10.1200/JCO.2008.18.7229 19064967

[B23] Larmonier N Marron M Zeng Y Tumor-derived CD4(+)CD25(+) regulatory T cell suppression of dendritic cell function involves TGF-beta and IL-10 Cancer Immunol Immunothe 2007 56 48 59 10.1007/s00262-006-0160-8 PMC11030031 16612596

[B24] Ghiringhelli F Menard C Martin F Zitvogel L The role of regulatory T cells in the control of natural killer cells: relevance during tumor progression Immunol Rev 2006 214 229 238 10.1111/j.1600-065X.2006.00445.x 17100888

[B25] Lim HW Hillsamer P Banham AH Kim CH Cutting edge: direct suppression of B cells by CD4+ CD25+ regulatory T cells J Immunol 2005 175 4180 83 16177055 10.4049/jimmunol.175.7.4180

[B26] Raoul JL Van Laethem JL Peeters M Cetuximab in combination with irinotecan/5-fluorouracil/folinic acid (FOLFIRI) in the initial treatment of metastatic colorectal cancer: a multicentre two-part phase I/II study BMC Cancer 2009 9 112 10.1186/1471-2407-9-112 19366444 PMC2678147

[B27] Tabernero J Van Cutsem E Díaz-Rubio E Phase II trial of cetuximab in combination with fluorouracil leucovorin, and oxaliplatin in the first-line treatment of metastatic colorectal cancer J Clin Oncol 2007 25 5225 32 10.1200/JCO.2007.13.2183 18024868

[B28] Therasse P Arbuck SG Eisenhauer EA New guidelines to evaluate the response to treatment in solid tumors. European Organization for Research and Treatment of Cancer. National Cancer Institute of the United States. National Cancer Institute of Canada J Natl Cancer Inst 2000 92 205 16 10.1093/jnci/92.3.205 10655437

[B29] Heo DS Park JG Hata K Day R Herberman RB Whitesite T Evaluation of Tetrazolium-based semiautomatic colorimetric assay for measurement of human antitumor cytotoxicity Cancer Res 1990 50 3681 3690 2340518

[B30] Van de Losdrecht A Cell mediated cytotoxicity against U 937 cells by human monocytes and macrophages in a modified colorimetric MTT assay. A methodological study. J Immunol Methods 1991 141 15 22 10.1016/0022-1759(91)90205-T 1865120

[B31] Cartron G Dacheux L Salles G Solal-Celigny P Therapeutic activity of humanized anti-CD20 monoclonal antibody and polymorphism in IgG Fc receptor Fc RIIIa gene Blood 2002 99 754 758 10.1182/blood.V99.3.754 11806974

[B32] Weng W-K Levy R Two immunoglobulin G fragment C receptor polymorphisms independently predict response to rituximab in patients with follicular lymphoma J Clin Oncol 2003 21 3940 3947 10.1200/JCO.2003.05.013 12975461

[B33] Musolino A Naldi N Bortesi B Immunoglobulin G fragment C receptor polymorphisms and clinical efficacy of trastuzumab-based therapy in patients with HER-2/neu-positive metastatic breast cancer J Clin Oncol 2008 26 1789 1796 10.1200/JCO.2007.14.8957 18347005

[B34] Carson WE Parihar R Lindemann MJ Interleukin-2 enhances the natural killer cell response to Herceptin-coated Her2/neu-positive breast cancer cells Eur J Immunol 2001 31 3016 25 10.1002/1521-4141(2001010)31:10<3016::AID-IMMU3016>3.0.CO;2-J 11592078

[B35] Lanier LL Kipps TJ Phillips JH Functional properties of a unique subset of cytotoxic CD3 ^+ ^T lymphocytes that express Fc receptors for IgG (CD16/Leu-11 antigen) J Exp Med 1985 162 2089 106 10.1084/jem.162.6.2089 2415663 PMC2187997

[B36] Lefebvre ML Krause SW Salcedo M Nardin A *Ex vivo*-activated human macrophages kill chronic lymphocytic leukemia cells in the presence of rituximab: mechanism of antibody-dependent cellular cytotoxicity and impact of human serum J Immunother 2006 29 388 97 10.1097/01.cji.0000203081.43235.d7 16799334

[B37] Stockmeyer B Beyer T Neuhuber W Polymorphonuclear granulocytes induce antibody-dependent apoptosis in human breast cancer cells J Immunol 2003 171 5124 9 14607911 10.4049/jimmunol.171.10.5124

[B38] Arnould L Gelly M Penault-Llorca F Trastuzumab-based treatment of HER2-positive breast cancer: An antibody-dependent cellular cytotoxicity mechanism? Br J Cancer 2006 94 259 67 10.1038/sj.bjc.6602930 16404427 PMC2361112

[B39] Manches O Lui G Chaperot L In vitro mechanisms of action of rituximab on primary non-Hodgkin lymphomas Blood 2003 101 949 54 10.1182/blood-2002-02-0469 12393572

[B40] Barriere J Fischel J Formento P Cetuximab-mediated antibody-dependent cellular cytotoxicity (ADCC) against tumor cell lines characterized for EGFR expression and K-ras mutation ASCO Annual Meeting 2009 e14583

[B41] Greenwood J Clark M Waldmann H Structural motifs involved in human IgG antibody effector functions Eur J Immunol 1993 23 1098 1104 10.1002/eji.1830230518 8477804

[B42] Schleypen JS Baur N Kammerer R Cytotoxic markers and frequency predict functionnal capacity of natural killer cells infiltrating renal carcinoma Clin Cancer Res 2006 12 718 25 10.1158/1078-0432.CCR-05-0857 16467081

[B43] Carrega P Morandi B Costa R Natural killer cells infiltrating human nonsmall-cell lung cancer are enriched in CD56 Bright CD16 - cells and display an impaired capability to kill tumor cells Cancer 2008 112 863 75 10.1002/cncr.23239 18203207

[B44] Gennari R Menard S Fagnoni F Pilot study of the mechanism of action of preoperative trastuzumab in patients with primary operable breast tumors overexpressing Her2 Clin Cancer Res 2004 10 5650 5655 10.1158/1078-0432.CCR-04-0225 15355889

