# Supplementary material for: Putative contribution of CD56 positive cells in cetuximab treatment efficacy in first-line metastatic colorectal cancer patients
Source: BMC Cancer. 2010 Jun 30;10:340. doi: 10.1186/1471-2407-10-340 (PMC2912265; doi:10.1186/1471-2407-10-340)
Supplement: Additional file 1 — Supplemental Table S1. Summary of the immunohistochemistry. [file 1471-2407-10-340-S1.DOC]

**ADDITIONAL FILES**

**Putative contribution of CD56 positive cells in cetuximab treatment efficacy in first-line metastatic colorectal cancer patients**

Raphaël Maréchal^1,2^, Jef De Schutter^3^, Nathalie Nagy ^4^, Pieter Demetter ^4^, Arnaud Lemmers^5^, Jacques Devière^6^, Isabelle Salmon^4^, Sabine Tejpar^7^ , Jean-Luc Van Laethem^1^

**Additional file 1:**

**Title: Supplemental Table S1**

**Description: Summary of the immunohistochemistry.**

| **Primary antibody** | **Marker/Clone** | **Supplier** | **Mono(M), poly(P)** | **dilution** | **Antigen retrieval** |
| --- | --- | --- | --- | --- | --- |
| CD3  CD4  CD8  CD56  CD68  Foxp3  EGFR | PS1  4B12  C8/144B  123C3D5  KP1  236A/E7  31G7 | Novocastra  Neomarkers  DAKO  Neomarkers  DAKO  Abcam  Zymed | M  M  M  M  M  P  M | 1/100  1/50  1/200  1/50  1/800  1/100  1/100 | MW, EDTA  MW, citrate  MW, citrate  MW, citrate  MW, citrate  MW,EDTA  Proteinase K |

MW: microwave
