# Supplementary material for: Putative contribution of CD56 positive cells in cetuximab treatment efficacy in first-line metastatic colorectal cancer patients
Source: BMC Cancer. 2010 Jun 30;10:340. doi: 10.1186/1471-2407-10-340 (PMC2912265; doi:10.1186/1471-2407-10-340)
Supplement: Additional file 2 — Description of the MTT assay. [file 1471-2407-10-340-S2.DOC]

**Additional file 2:**

**Title: Description of the MTT assay**

After incubation, all non adherent cells present in the wells were removed by performing three consecutive washes with culture medium and 50μl of MTT (Sigma) solution (5mg/ml) was added to each well followed by 150 μl culture medium and the plates were incubated for additional 4h. Solubilization of formazan crystals formed in viable cells were achieved with isopropanol and the absorbance was read at a wavelength of 540nm. The percentage of cell death was calculated as [(A – B) – ( C – D )/ A] x 100 where A is the optimal optical density (A_540_) of 5 10^4^ cells/well, B the A_540_ of non specifically detached tumor cells during washing which was estimated from A_540_ of 5x10^4^ tumor cells after washing 3 times, C is the experimental A_540_ of adherent tumor cells remaining in the wells after washing and D is the A_540_ of adherent effectors remaining in the control wells after washing.

Experiments conducted in a preliminary phase to select optimal conditions for the ADCC effect showed that *(a)* target cells were not killed after exposure to CTX in the absence of PBMC *(b)* 10µg/ml was the optimal CTX concentration for saturing ADCC assay by PBMC.
