# Supplementary material for: Putative contribution of CD56 positive cells in cetuximab treatment efficacy in first-line metastatic colorectal cancer patients
Source: BMC Cancer. 2010 Jun 30;10:340. doi: 10.1186/1471-2407-10-340 (PMC2912265; doi:10.1186/1471-2407-10-340)
Supplement: Additional file 3 — Supplemental Table S2. Cetuximab-based chemotherapy efficacy in WT tumors based on the presence or absence of tumor infiltrating CD56+ cells. [file 1471-2407-10-340-S3.DOC]

**Additional file 3:**

**Title: Supplemental Table S2**

**Description:** Cetuximab-based chemotherapy efficacy in WT tumors based on the presence or absence of tumor infiltrating CD56+ cells.

|  | KRAS WT (n=20) | KRAS WT and CD56+ (n=10) | KRAS WT and CD56- (n=10) | p-value |
| --- | --- | --- | --- | --- |
| OR | 13/20 (65%) | 8/10 (80%) | 5/10 (50%) | 0.108 |
| PFS, months  median  (95% CI) | 9.2  (4.9-10.04) | 11.17  (7.3-15.04) | 5.4  (3.08-7.72) | 0.35 |

Abbreviations: OR: Overall response, PFS: Progression-Free Survival, CI: Confident Interval, WT: Wild-Type
